# Evolutionary Dynamics of Dosage Compensation and Sex-biased Gene Expression in Morabine Grasshopper *Vandiemenella viatica*

**DOI:** 10.1093/gbe/evag026

**Published:** 2026-02-04

**Authors:** Suvratha Jayaprasad, Holger Schielzeth, Octavio M Palacios-Gimenez

**Affiliations:** Population Ecology Group, Institute of Biodiversity, Ecology and Evolution, Friedrich Schiller Universität, Jena, DE 07743-, Germany; Population Ecology Group, Institute of Biodiversity, Ecology and Evolution, Friedrich Schiller Universität, Jena, DE 07743-, Germany; German Centre for Integrative Biodiversity Research (IDiv) Halle-Jena-Leipzig, Puschstraße 4 04103 Leipzig, Germany; Population Ecology Group, Institute of Biodiversity, Ecology and Evolution, Friedrich Schiller Universität, Jena, DE 07743-, Germany; German Centre for Integrative Biodiversity Research (IDiv) Halle-Jena-Leipzig, Puschstraße 4 04103 Leipzig, Germany; Department of Organismal Biology – Systematic Biology, Evolutionary Biology Centre, Uppsala University, Uppsala SE-75236, Sweden

**Keywords:** dosage compensation, sex-biased gene expression, X-linked genes, transcriptional regulation

## Abstract

Sex chromosome evolution and gene regulation are closely linked but remain understudied in many taxa. Young neo-sex chromosomes offer unique insights into these processes. We examine dosage compensation and sex-biased gene expression in *Vandiemenella viatica* grasshoppers by comparing the ancestral X chromosome in the P24X0 race with derived neo-sex chromosomes in the P24XY race. The P24XY neo-XY arose via X-autosome fusion: the XL arm represents the ancestral X and the XR arm a former autosome (chromosome 1 in P24X0) now part of the neo-X and homologous to the neo-Y. We first assess dosage compensation via male and female gene expression. In somatic tissues, male P24X0 X-linked and P24XY XL-linked genes are upregulated to match both female expression and autosomal levels, indicating near-complete dosage compensation. In testes, expression of X-linked and the XL-linked genes is reduced nearly 4-fold reflecting absent dosage compensation and the presence of meiotic X chromosome inactivation. We then analyze sex-biased gene expression across tissues and chromosomes. Gonads show stronger sex-biased gene expression than somatic tissues. Female-biased genes are concentrated on the P24X0 X and P24XY XL, whereas male-biased genes are enriched on autosomes and the XR arm of the neo-X. Overall, the ancestral X in P24X0 and the XL arm of the P24XY neo-X are hypertranscribed, while the XR arm retains autosomal expression, male-biased enrichment, and lacks dosage compensation. These patterns show that dosage compensation is regulated at levels of chromosome arms and illustrate how chromosome structure, gene regulation, and reproduction interact, shedding light on sex chromosome evolution in *V. viatica*.

Significance This study explores the evolution of sex chromosomes and gene regulation in *Vandiemenella viatica* grasshoppers, comparing the X chromosome in the P24X0 race with derived neo-XY sex chromosomes in the P24XY race. The analysis shows that dosage compensation occurs in non-reproductive tissues, where male X-linked genes are upregulated to match female and autosomal gene expression, but is absent in reproductive tissues, where X-linked genes appear to be meiotically silenced. The study identifies distinct patterns of sex-biased gene expression: female-biased genes are concentrated on the X chromosome and the XL arm of the neo-X, while male-biased genes are found on autosomes and the XR arm of the neo-X. The X (and XL arm) chromosome is thus feminized, as it is enriched with female-biased genes. These findings demonstrate how chromosome structure influences gene regulation and dosage compensation, providing new insights into the evolution of sex chromosomes.

## Introduction

Sex chromosomes across diverse clades show convergent features in structure and function, a phenomenon central to evolutionary biology (Charlesworth et al. 2005). Following their divergence from autosomal pairs, Y chromosomes typically undergo degeneration, becoming gene-poor and heterochromatic (Bachtrog 2013), whereas X chromosomes typically remain gene-rich and euchromatic (Vicoso and Charlesworth 2006). To counteract reduced gene expression caused by Y chromosome degeneration, the X often evolves dosage compensation (DC) mechanisms, to compensate for its haploid state in the heterogametic sex (Charlesworth 1998; Vicoso et al. 2013; Graves 2015; Duan and Larschan 2019). When ancestral expression levels are unknown, the equivalence of expression levels in females and males is often taken as evidence for DC, though some suggest calling this “dosage balance” and reserving DC for cases with known ancestral gene expression (Gu and Walters 2017).

DC mechanisms vary significantly among taxa. The most extensively studied DC process is X chromosome inactivation (XCI) in somatic tissues of female mammals (Lyon 1974). This process involves the transcriptional silencing of one of the two X chromosomes, effectively matching transcription to the single X chromosome present in males. The silencing is achieved through a formation of heterochromatin and the recruitment of various proteins and noncoding RNAs, leading to epigenetic modifications such as histone deacetylation and DNA methylation (Deng et al. 2011; Schulz and Heard 2013; Tukiainen et al. 2017). Some genes on the inactive X chromosome can escape this silencing, resulting in their expression at varying levels, which adds complexity to the regulation of X-linked genes (Carrel and Willard 2005; Disteche 2012; Tukiainen et al. 2017). In *Drosophila* somatic tissues, DC is achieved by increasing the transcription of genes on the single X chromosome in males to match the expression levels of the two X chromosomes in females (Lucchesi and Kuroda 2015). The process is mediated by the DC complex, which binds to the X chromosome and promotes gene transcription (Lucchesi and Kuroda 2015). Similarly, in *Caenorhabditis elegans* somatic tissues, both X chromosomes in hermaphrodites undergo transcriptional downregulation, while in males, the single X chromosome is upregulated, overall achieving equal expression of X-linked genes (Disteche 2012).

A distinct form of X-inactivation occurs in males during spermatogenesis as germ cells enter meiosis. This process is known as meiotic sex chromosome inactivation (MSCI) (Cloutier and Turner 2010; Turner 2015) and has become an important focus in the study of epigenetic gene regulation. In a variety of organisms, including mammals (Khil et al. 2004), *C. elegans* (Kelly et al. 2002), *Drosophila* (Mahadevaraju et al. 2021), and grasshoppers (Viera et al. 2021), X chromosome expression is reduced during spermatogenesis. MSCI is well-characterized in mammals, with early transcriptional silencing of the X and Y chromosomes during mid-spermatogenesis (Turner 2015). During this stage, both chromosomes condense into a heterochromatic “XY body” within the pachytene spermatocyte nucleus. This inactivation is believed to protect the dissimilar chromosomes by shielding unsynapsed regions from potentially harmful transposons, which are not recognized as foreign in the absence of a homolog (Huynh and Lee 2005). Additionally, this inactivation may prevent undesired recombination between the X and Y chromosomes or facilitate DNA repair in the absence of recombination (Lu and Yu 2015). In some cases, it may also serve to mark the paternal X chromosome for future imprinting (McKee et al. 2012).

Even in species with established DC, the X chromosome often carries a surplus of sex-biased genes, suggesting the evolution of specialized gene content. The direction of this bias varies among taxa: in flies and nematodes, the X chromosome is feminized (Parisi et al. 2003; Albritton et al. 2014; Vicoso and Bachtrog 2015) whereas in mammals, it shows a masculinized gene profile (Lercher et al. 2003). The highly specialized biological functions of fully differentiated sex chromosomes is believed to prevent their reversion to autosomes (Bull 1983; Pokorná and Kratochvíl 2009). Consequently, species with fully differentiated sex chromosomes rarely undergo sex chromosome turnover (the emergence of a new XY pair or the reversal of former X or Y chromosomes to an autosomal state). Differentiated sex chromosomes are thus regarded as “evolutionary traps”. This view is well-supported in eutherian mammals (Waters et al. 2007), snakes (Matsubara et al. 2006), and birds (Shetty et al. 1999). However, the situation seems to be different in insects. Dipteran flies, for example, have experienced multiple instances of turnover involving the ancestral differentiated X (Vicoso and Bachtrog 2015). Likewise, the observed high rate of Y chromosome loss and gain in beetles suggests frequent sex chromosome turnover within this clade (Blackmon and Demuth 2014). Whether these cases represent exceptions or if the frequency of turnover has been underestimated in other clades with differentiated sex chromosomes remains unclear.

Comparative analyses of ancient versus nascent sex chromosomes will be critical for understanding the evolutionary dynamics of DC. The wingless matchstick Australian morabine grasshoppers of the *Vandiemenella viatica* species group (hereafter referred to as the *viatica* group) offer a compelling model. The *Vandiemenella* genus includes 2 nominal species (*V. pichirichi* and *V. viatica*) as well as 11 provisional chromosomal races, which are cytogenetically and morphologically distinguishable: *viatica*17, *viatica*19, P24X0, P24XY, P24XY-translocation, P25X0, P25XY, P45bX0, P45bXY, P45cX0, and P50X0 (White 1978). The observed cytogenetic differences are caused by extensive chromosome rearrangements, including centric fusions, fissions, translocations, and inversions (White et al. 1967, 1969; White 1978; Mrongovius 1979). Most of the chromosomal races show males with an X0 sex chromosome configuration and females with XX sex chromosomes (White 1978). However, three of the known races (P24XY, P25XY, and P45bXY) involve fusions between the ancestral X chromosome and autosomes, leading to three independently evolved cases of neo-XY/neo-XX chromosome systems (White 1978; Palacios-Gimenez et al. 2020; Jayaprasad et al. 2024).

The meiotic behavior of the X chromosome in the X0 races is readily identifiable as it exists as a single acrocentric, unpaired, and achiasmatic (non-recombining) element (White et al. 1967). In the P24XY race, the neo-X is metacentric, with an XL arm derived from the ancestral X chromosomes and an XR arm derived from an autosome (chromosome 1 in X0 races) involved in a centric fusion. The neo-Y is acrocentric and homologous to the XR arm (White 1973). During male meiosis, the neo-Y pairs with the XR arm, typically forming a single chiasma that resolves before the first metaphase (White et al. 1967). The XL arm is hemizygous in males like the ancestral X in the X0 races. As in other orthopterans (White 1973; Hewitt 1979; Castillo et al. 2010), heteropycnosis is a common feature of morabine sex chromosomes during cell division, particularly in spermatogenesis. In spermatogonial metaphases (mitotic cells), the original unpaired X chromosome (or the XL arm in the neo-X) shows negative heteropycnosis, appearing less condensed. In contrast, the XR arm of the neo-X and the neo-Y retain their autosomal characteristics, remaining non-heteropycnotic (White et al. 1967). During prophase I of meiosis, however, the unpaired X chromosome (or XL arm in the neo-X) undergoes positive heteropycnosis, becoming more condensed and forming the so-called sex chromatin (White et al. 1967). Meanwhile, the XR arm of the neo-X and the neo-Y remain faithful to their autosomal origin by staying non-heteropycnotic, as seen in spermatogonial metaphases. The heteropycnotic behavior of orthopteran sex chromosomes has long been interpreted as MSCI (White 1973; Hewitt 1979).

The unique sex chromosome variation in morabine grasshoppers allows for independent comparisons between the ancestral X0 and the derived neo-XY sex chromosome systems, facilitating investigations into the early evolution of sex chromosome expression patterns. Although the molecular basis of DC in grasshoppers is unknown, its role would be the restoration of sex-linked gene expression in the heterogametic sex (males) to ancestral, diploid levels (i.e. achieve DC) while also ensuring equal expression of sex chromosomes between the sexes (i.e. dosage balance), despite males possessing only a single X chromosome (Smith et al. 2014; Mahajan and Bachtrog 2015; Gu and Walters 2017). A unique feature in neo-sex chromosome systems is that the neo-XR shares homology with the neo-Y. Although sex chromosomes tend to decay and to lose genes, many of the genes are still present on both gametologs (Ming et al. 2021; Nozawa et al. 2021; Huang et al. 2022). This sets young neo-sex chromosomes systems apart from ancient, degraded sex chromosomes (as in *Drosophila*, mammals and birds), since we can test if the fused chromosome retains its autosomal characteristics in gene expression or conforms to the expression pattern of the (ancestral) X chromosomes.

**Fig. 1.**
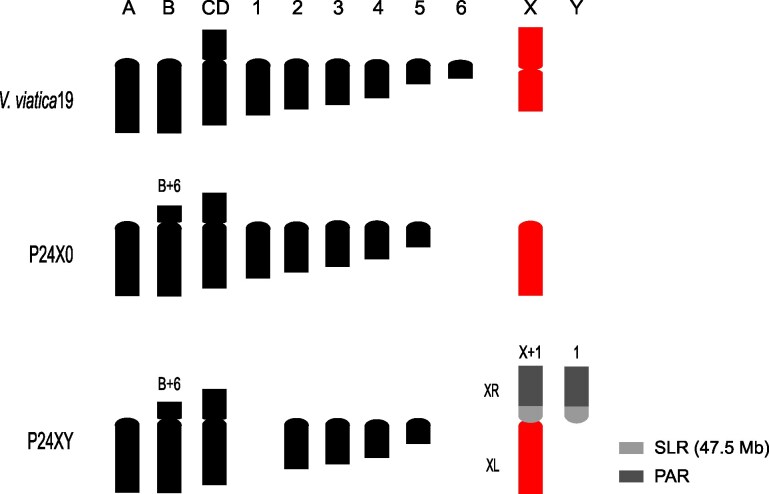
Schematic drawing of male haploid karyotypes and hypothesized chromosomal changes of the morabine grasshoppers *V. viatica*19, P24X0 and P24XY. The karyotype of the race *V. viatica*19 is considered ancestral (White 1978). Chromosomes are sorted by size and centromere position. Note the metacentric (bi-armed) X chromosome in *V. viatica*19 and the acrocentric (uni-armed) X in P24X0, resulted from an inversion involving the centromere. The neo-XY sex chromosome formed via centric fusion between the ancestral X chromosome (red) and one of the autosomes (chromosome 1). XL refers to the arm originating from the ancestral X chromosome fused to an autosome, while XR designates the derived autosomal arm of the neo-X that shares homology with the neo-Y. The neo-XY sex-liked region (SLR) and the pseudoatusosomal region (PAR) are defined by colors, based on previous results (Jayaprasad et al. 2024).

Here, we reanalyze publicly available data to explore the biology of the X chromosome in the *viatica* group, focusing on two chromosomal races: P24X0 and P24XY (Fig. 1). The two races have diverged around 1.45 Mya (Jayaprasad et al. submitted). Both races have been sequenced at the DNA and RNA level (Palacios-Gimenez et al. 2020; Jayaprasad et al. 2024). Our analysis of chromosome-level assemblies and RNA-seq data from both males and females shows that ancestral X-linked genes show similar expression levels between the sexes in nonreproductive tissues, despite being present as a single copy in males, aligning with the predictions of dosage DC. In reproductive tissues, however, we find no evidence of DC and the near 4-fold reduction of X- and XL-linked gene expression suggests MSCI in male testes. In contrast, the autosomes incorporated into the newly evolved neo-XY system show gene expression levels comparable to autosomes regardless of the sex or tissue analyzed, thus indicating stable gene expression pattern following the X-A fusion. The proportion of female-biased gene expression in gonads was higher on the ancestral X (and/or XL) chromosome compared to autosomes, indicating a feminized gene content of the ancestral X (and/or XL arm) chromosome. Downregulation of the ancestral X-linked genes in males may have evolved as a mechanism to mitigate the effects of the X chromosome and/or facilitate the transcriptional silencing of unsynapsed X chromatin in the testes.

## Results

### Dosage Compensation Across Tissues

The sex chromosomes in races P24X0 and P24XY (including the 47.5 Mb P24XY neo-XY sex-linked region) have been previously identified in genome assemblies using whole-genome sequencing data by analyzing sex-specific differences in genome read coverage and heterozygosity (Jayaprasad et al. 2024). To investigate whether any DC mechanisms have evolved in morabine grasshoppers, we analyzed RNA-seq data from heads, legs, ovaries, and testes of P24X0 and P24XY males and females to estimate male and female gene expression levels for each race and tissue (5 to 11 individuals per tissue/sex/race). Reads were mapped to their respective chromosome-level assemblies to quantify expression levels of X-linked and autosomal genes. For P24X0, we analyzed genes separately on autosomes, chromosome 1 (homologous to the neo-XR arm and neo-Y in P24XY) and the X chromosome. For P24XY, we analyzed genes separately on autosomes, genes on the XL arm of the neo-X chromosome (homologous to the X in P24X0), and genes on neo-XY sex-linked region (SLR) and pseudoautosomal region (PAR) from the XR-Y arms, following the chromosome/arm descriptions (Fig. 1). Dividing the XR-Y into SLR and PAR enabled us to compare gene expression evolution in regions with ongoing recombination (PAR) versus those where recombination ceased relatively recently (SLR). The boundaries delimiting the PAR and SLR have been obtained from a previous publication (Jayaprasad et al. 2024). After removing lowly expressed genes (retaining TPM > 1) and considering only genes expressed in both males and females, we analyzed 656 X-linked genes and 6,367 autosomal genes (665 on chr1 and 5,702 on other autosomes) in P24X0, and 1,139 X-linked genes (540 on the XL arm, and 523 on XR-Y PAR and 76 on XR-Y SLR) and 5,203 autosomal genes in P24XY. We then compared the male-to-female ratio of X-linked gene expression to that of autosomal genes. With complete DC, we expected the male-to-female expression ratio of X-linked genes to be close to 1 and overall expression comparable to autosomal genes. Without DC, we expect half the expression levels of X-linked genes in males as compared to females and as compared to autosomes. MSCI would be indicated if male expression is reduced to less than half in the testes.

**Fig. 2.**
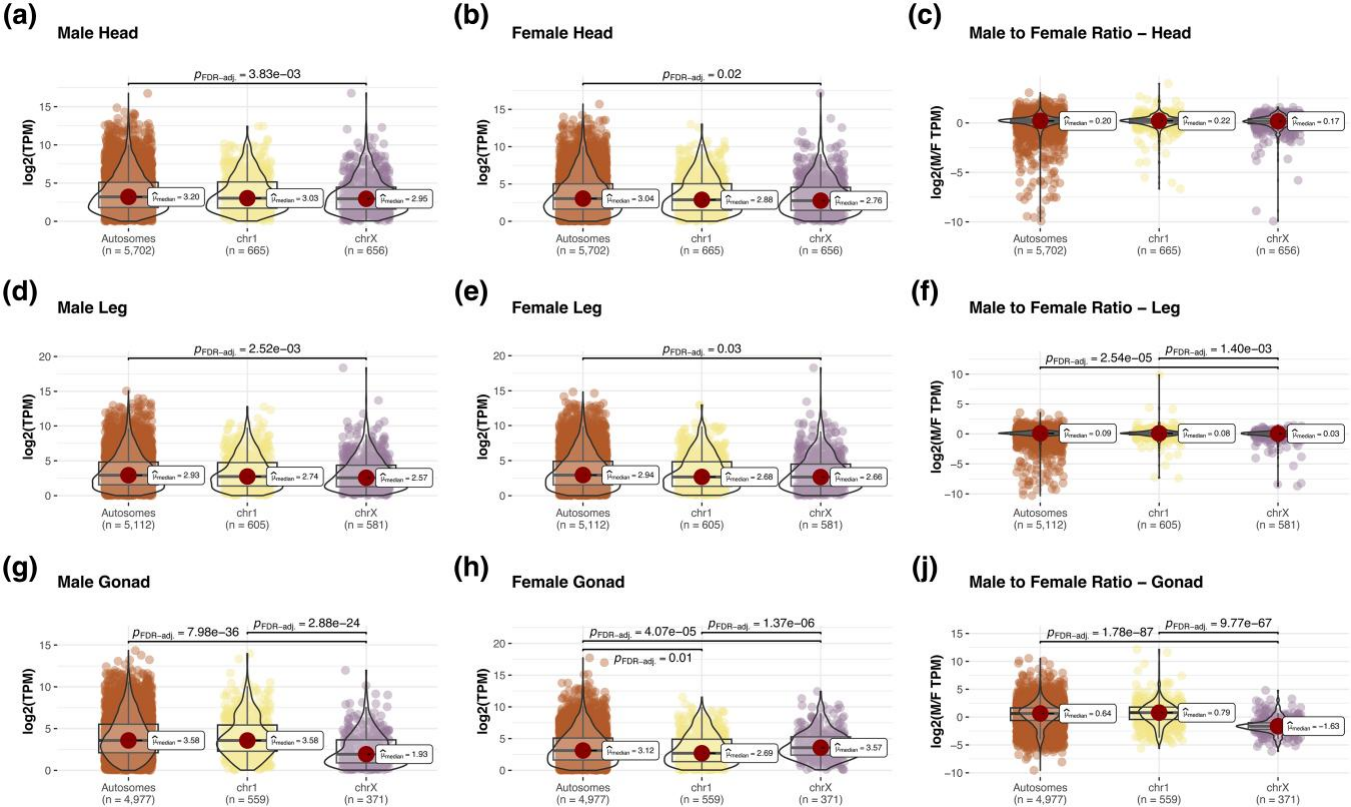
Dosage compensation (a–i) in P24X0. A combination of box and violin plots along with jittered data points with statistical details included as a subtitle. Chromosome 1 = chr1; chromosome X = chrX. The chr1 is plotted separately from the other autosomes (Autosomes) for comparison, as it is part of the X-A fusion in the P24XY race. The *n* values for each chromosome type indicate the number of genes analyzed after retaining those with TPM > 1. The median values (${\hat{\mu }}_{median}$) for each group are indicated. *P*_FDR-adj_ indicates *P*-values after Benjamini−Hochberg adjustment for multiple comparisons.

**Fig. 3.**
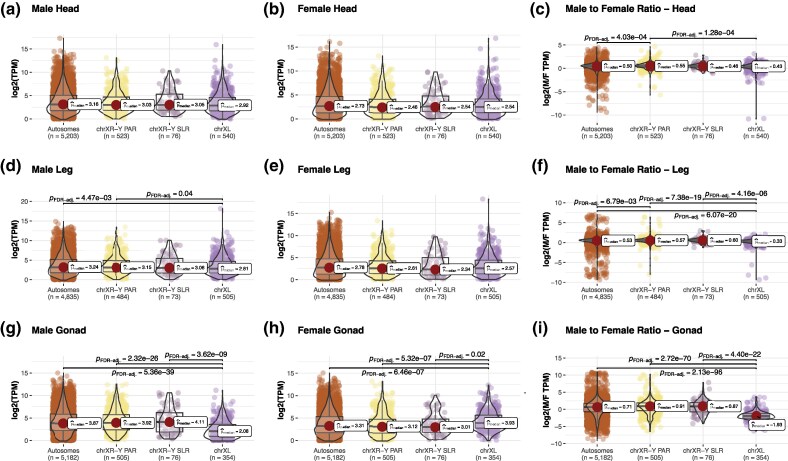
Dosage compensation (a–i) in P24XY. A combination of box and violin plots along with jittered data points with statistical details included as a subtitle. ChrXL is the ancestral X (XL arm) of the neo-X, while chrXR-Y represents the fused chr1 (XR arm) of the neo-X, and -Y the neo-Y (homolog to the XR arm). The chrXR-Y is divided into a sex-linked region (SLR) and a pseudoautosomal region (PAR), based on previous results (Jayaprasad et al. 2024). The *n* values for each chromosome type indicate the number of genes analyzed after retaining those with TPM > 1. The median values (${\hat{\mu }}_{median}$) for each group plot are indicated. *P*_FDR-adj_ indicates *P*-values after Benjamini−Hochberg adjustment for multiple comparisons.

For P24X0, our analysis revealed notable differences in expression levels between X-linked and autosomal genes across tissues and sexes (Fig. 2). In head and leg tissues, X-linked genes showed a male-to-female expression ratio close to that of autosomal genes, suggesting near complete DC for the single X in males. However, in gonadal tissues (ovaries and testes), X-linked genes showed 4-fold lower gene expression in males relative to females and to autosomes (comparing the median values), suggesting the absence of DC and the presence of MSCI. These findings illustrate tissue-specific DC in X-linked gene expression, with X-upregulation evident only in nonreproductive tissues and X-downregulation/inactivation in testis. The pattern was highly similar for the P24XY race, in which male-hemizygous XL (homologous to X in P24X0) genes of the neo-X were upregulated only in nonreproductive tissues and downregulated/inactivated in testis (Fig. 3). In contrast, genes on the XR-Y SLR and on the XR-Y PAR were expressed to a similar level as autosomal genes, indicating that no chromosome-wide DC has evolved in males. This suggests that DC is specific to the XL arm of the neo-X rather than the entire chromosome.

### Expression Profiles of X-linked Genes that Have One-to-One Orthologs Across P24X0 and P24XY

**Fig. 4.**
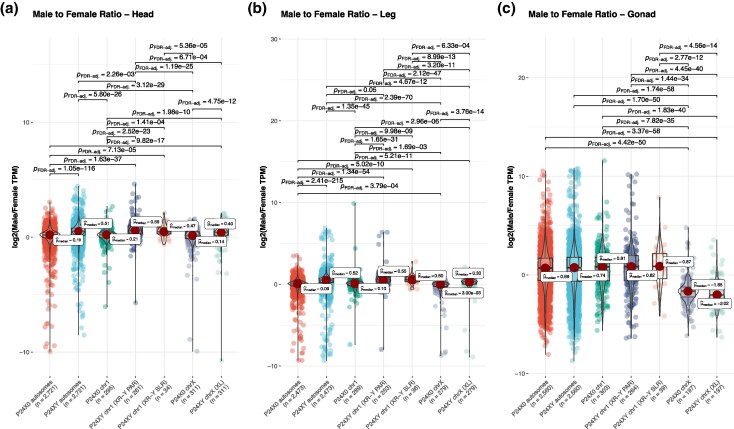
Comparison of expression levels of one-to-one orthologous genes (a–c) from P24X0 and P24XY. A combination of box and violin plots along with jittered data points with statistical details included as a subtitle. Chromosomal annotations as in Figs. 2 and 3. The *n* values for each chromosome type indicate the number of genes analyzed after retaining those with TPM > 1. The median values (${\hat{\mu }}_{median}$) for each group are indicated. *P*_FDR-adj_ indicates *P*-values after Benjamini−Hochberg adjustment for multiple comparisons.

To investigate changes in gene expression across-tissue during sex chromosome evolution, we compared expression profiles of one-to-one orthologs between P24X0 and P24XY (Fig. 4). This approach has previously been used to study sex chromosome gene expression in *Drosophila* (Nozawa et al. 2021, 2016) and mammals (Lin et al. 2012). Orthologs were identified via bidirectional best BLASTp hits (e-value < 1e-10) across autosomes (AA:AA), chromosome 1 and the XR arm (chr1:XR), and chromosome X and chromosome XL (X:XL). In P24XY, the XR arm was separated into the SLR and PAR to reflect their differing evolutionary dynamics. While the SLR show signs of degeneration (Jayaprasad et al. 2024) and potentially altered expression pattern (due to the occurrence of X-specific alleles), the PAR continues to recombine and should behave more like autosomes. In P24X0, the chr1—homolog to the XR arm of the P24XY neo-X—remains largely recombining (Jayaprasad et al. 2024).

The chr1 and the XR arm share 303 one-to-one orthologs. The expression patterns of one-to-one orthologs between P24X0 and P24XY are consistent with those observed at the intra-race level. We found that the median expression ratio for the hemizygous X:XL to be ∼1, similar to AA:AA and chr1:XR in somatic tissues, indicating chromosome-wide DC for X-linked genes in the soma (Fig. 4a–b). In contrast, we found 4-fold reduction in the median X:XL expression ratio compared to AA:AA and chr1:XR in testis, supporting the absence of DC and the presence of MSCI for X-linked genes in testis (Fig. 4c).

### Sex-biased Gene Expression

We investigated the genomic distribution of sex-biased genes in various tissues of the P24X0 and P24XY races (Table 1). To encompass the full spectrum of expression levels, the analysis was performed at the intra-race level rather than being limited to one-to-one orthologous genes. The proportion of sex-biased genes was highest in gonads and lowest in nonreproductive tissues. This pattern was consistent across both P24X0 and P24XY races (Table 1).

**Table 1:** Numbers of sex-biased genes per chromosome in P24X0 and P24XY. SLR stands for sex-linked region, and PAR stands for pseudoautosomal region

P24X0						
Tissue	Chromosome	Total genes	Expressed genes	Male-biased genes (%)	Un-biased genes (%)	Female-biased genes (%)
Head	chrX	1578	1228	0 (0)	1226 (98)	2 (0.2)
…	chr1	1415	1162	4 (0.3)	1158 (99.7)	0 (0)
…	Autosomes	12448	10135	2 (0.02)	10132 (99.97)	1 (0.01)
Leg	chrX	1578	1129	0 (0)	1126 (99.7)	3 (0.3)
…	chr1	1415	1065	3 (0.3)	1062 (99.7)	0 (0)
…	Autosomes	12448	9332	2 (0.02)	9325 (99.93)	5 (0.05)
Gonad	chrX	1578	1172	14 (1.2)	1014 (86.5)	144 (12.3)
…	chr1	1415	1131	99 (8.7)	977 (86.6)	55 (4.7)
…	Autosomes	12448	9928	767 (7.7)	8560 (86.3)	601 (6)

P24XY						
Tissue	Chromosome	Total genes	Expressed genes	Male-biased genes (%)	Un-biased genes (%)	Female-biased genes (%)
Head	chrXL	1510	1047	0 (0)	1045 (99.8)	2 (0.2)
…	chrXR-Y PAR	1188	926	0 (0)	926 (100)	0 (0)
…	chrXR-Y SLR	248	179	0 (0)	179 (100)	0 (0)
…	Autosomes	12618	9725	0 (0)	9719 (99.9)	6 (0.7)
Leg	chrXL	1510	967	0 (0)	965 (99.8)	2 (0.2)
…	chrXR-Y PAR	1188	818	2 (0.3)	816 (99.7)	0 (0)
…	chrXR-Y SLR	248	148	0 (0)	148 (100)	0 (0)
…	Autosomes	12618	8639	2 (0.02)	8628 (99.88)	9 (0.1)
Gonad	chrXL	1510	1029	16 (1.5)	815 (79.3)	198 (19.2)
…	chrXR-Y PAR	1188	920	92 (10)	769 (83.6)	59 (6.4)
…	chrXR-Y SLR	248	177	18 (10.2)	148 (83.6)	11 (6.2)
…	Autosomes	12618	9827	893 (9.1)	8211 (83.6)	723 (7.3)

**Fig. 5.**
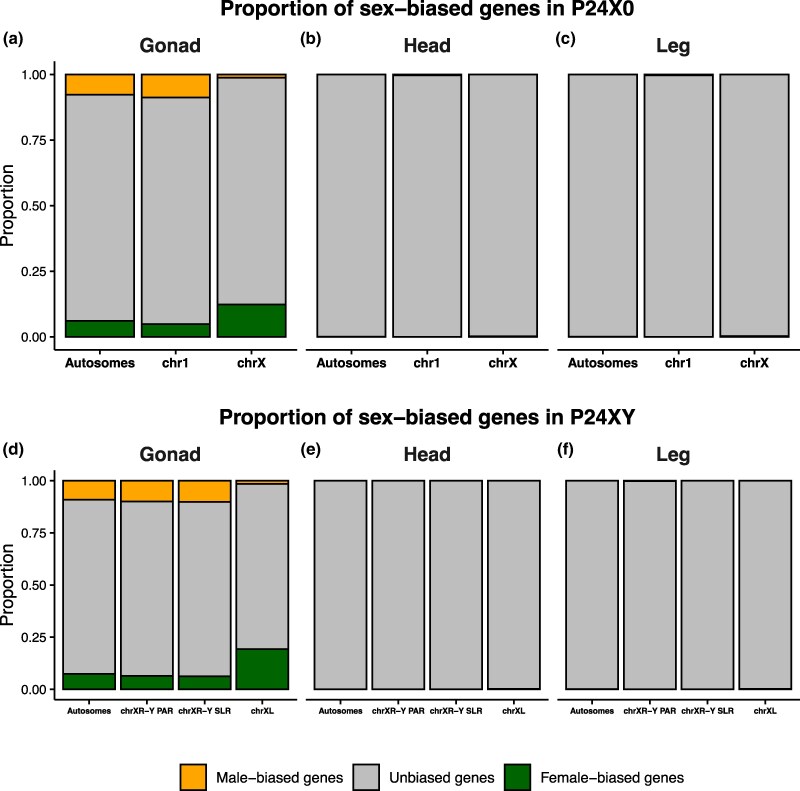
Proportion of male- and female-biased genes per tissue and chromosome type for P24X0 (a–c) and P24XY (d–f). Sex-biased gene expression was operationally defined log2FC < −2 or log2FC > 2 and adjusted *P*-value < 0.05. Genes that do not fit into this are classified as unbiased genes. Chromosomal annotations as in Figs. 2 and 3.

In the P24X0 race, we identified a total of 1,680 sex-biased genes in the gonads, compared to only 22 in the head and legs combined (Table 1). In the gonads, sex-biased genes were distributed unevenly across chromosomes. On the X chromosome, 12.3% of the expressed genes were female biased, whereas only 1.2% were male biased. In contrast, on autosomes 7.7% of expressed genes were male-biased and 6% were female-biased, indicating marginally higher proportion of male-biased genes on autosomes (Table 1, Fig. 5a). On chromosome 1, 8.7% of expressed genes were male-biased and 4.7% were female-biased. In somatic tissues, sex-biased expression was rare. In the head, we identified nine sex-biased genes, six of which were male biased and three female-biased. Notably, both sex-biased genes on the X chromosome were female-biased (0.2% of expressed X-linked genes), whereas all male-biased genes were autosomal or chromosome-1 linked (Table 1, Fig. 5b). A similar pattern was found in the leg, where 13 genes showed sex-biased expression. Of these, eight were female-biased and five were male-biased, and again all X-linked sex-biased genes (0.3% of expressed genes) were female-biased (Table 1, Fig. 5c). Overall, these patterns suggest that chromosome X plays a central role in mediating sex-biased expression, particularly in gonadal tissues.

In the P24XY race, we identified 2,010 sex-biased genes in the gonads, and 23 in the head and legs combined (Table 1). Of the gonadal sex-biased genes, 51% were female-biased. The female-bias was particularly pronounced on the XL arm of the neo-X chromosome, where 19.2% of expressed genes were female-biased and only 1.5% were male-biased. In contrast, both the XR-Y SLR and the XR-Y PAR showed a predominance of male-biased genes: on the XR-Y PAR, 10% of expressed genes were male biased compared with 6.4% female biased, and on the XR-Y SLR, 10.2% were male-biased versus 6.2% female-biased genes (Table 1, Fig. 5d). Autosomes also showed a modest excess of male-biased genes (9.1%) relative to female-biased genes (7.3%) (Table 1, Fig. 5d). As in P24X0, sex-biased expression in somatic tissues was limited. In the head, we identified eight sex-biased genes, all of which were female biased; two located on the XL arm of the neo-X, corresponding to 0.2% of expressed XL-linked genes, and six located on autosomes (0.07% of the expressed autosomal genes; Table 1, Fig. 5e). In the leg, we identified 15 sex-biased genes: two female-biased on the XL (0.2%), two male-biased on the XR-Y PAR (0.3%), and 11 autosomal genes (nine female-biased [0.1%] and two male-biased [0.02%], Table 1, Fig. 5f). These findings highlight the similarity in female-biased gene content between the ancestral X and its homolog in the fused state (XL) when comparing the P24X0 and P24XY races. Furthermore, it shows that the fused XR chromosome (irrespective of the SLR or PAR part) has similar sex-specific gene content as its homolog chr1 in the unfused state.

## Discussion

Our findings provide insights into the evolution of DC and sex-biased gene expression in *Vandiemenella* grasshoppers. The comparison of gene expression profiles between males and females in P24X0 and P24XY races reveals distinct X-linked gene regulation patterns. In nonreproductive tissues, DC occurs through upregulation of male X- and XL-linked genes, resulting in a balance of autosomal and X-linked gene expression. In the testis, male X- and XL-linked gene expression are dramatically downregulated, most likely by a combination of lack of DC and MSCI. These patterns demonstrate tissue-specific gene regulation. Furthermore, the analysis of sex-biased gene expression shows that the X (and XL arm) chromosome is enriched in female-biased genes, while the fused XR of the neo-X in P24XY show a gene expression pattern similar to its ancestral state as an autosome (represented by chromosome 1 in P24X0). This illustrates that the (ancestral) X chromosome is feminized in *Vandiemenella* grasshoppers, which probably represents a general state in grasshopper X chromosomes. DC and sex-biased gene expression are plausibly tailored to the specific requirements of each tissue, balancing gene expression in somatic cells and downregulating the feminized X chromosome in male-specific reproductive tissues.

The pattern of DC is consistent with DC in other insects, such as *Drosophila* (Lucchesi and Kuroda 2015), true bugs (Pal and Vicoso 2015), beetles (Mahajan and Bachtrog 2015), and stick insects (Djordjevic et al. 2025), where male X-linked genes are upregulated to achieve equal expression with females. Notably, DC seems to be more complete in morabine grasshoppers than in *Drosophila* (Mahadevaraju et al. 2021), since the balance is very closely equalized in our data. Given that RNA-seq data indicate that X- and XL-linked genes in morabine grasshoppers are expressed similarly in males and females, this likely reflects ancestral expression of the chromosomal arms (as exemplified by the expression profiles of one-to-one ortholog genes), as these chromosomes correspond to the ancestral X in many insects (Toups and Vicoso 2023; Jayaprasad et al. 2024; Li et al. 2024).

We found that male X- and XL-linked gene expression was dramatically reduced in testis compared to ovaries and nonreproductive tissues. The tissue sample from the testis represents a mixture of somatic, though male-specific, reproductive tissue and meiotically active cells during spermatogenesis. The pattern of 4-fold reduction in X- and XL-linked genes expression in testis cannot be solely explained by the absence of DC, thus providing evidence of MSCI. This aligns with findings in the grasshopper *Eyprepocnemis plorans*, where X inactivation begins in spermatogonia before meiosis, accompanied by persistent H3K9me3 heterochromatinization (Santos et al. 2024). MSCI is widespread in organisms with differentiated sex chromosomes (Cloutier and Turner 2010), though its timing varies. In mammals, MSCI silences both X and Y chromosomes during pachytene via extensive chromatin remodeling (Turner 2007, 2015; Santos et al. 2024). While the exact mediator(s) for MSCI in morabine grasshoppers are unknown, a mechanism similar to *E. plorans* seems most plausible.

MSCI represents a case of meiotic silencing of unsynapsed chromatin (MSUC), which prevents aneuploidy by silencing unpaired chromosomes (Turner 2007, 2015; Santos et al. 2024). Notably, MSCI is absent on the XR-Y chromosomes (both SLR and PAR) of P24XY that are partially nonrecombining, supporting MSUC for hemizygous X- and XL-linked genes and suggesting a chromosome arm-specific regulatory mechanism. MSCI of the X and XL chromosomes in morabine grasshoppers may help to counteract the feminizing effects of female-biased genes on the X and XL chromosomes, thereby preventing transcriptional conflicts during male germ cell development and ensuring proper meiotic processes.

The lack of MSCI in P24XY XR-Y chromosomes contrasts with MSCI of the achiasmatic XY in *D. melanogaster* and the largely nonrecombining XY in mammals. In *D. melanogaster*, X-linked genes are upregulated in the male germline (the canonical DC like in soma [Meiklejohn et al. 2011]) but dramatically silenced upon entry to meiosis (by MSCI), while the Y reaches peak transcriptional activity during meiosis (Mahadevaraju et al. 2021). In mammals, both X and Y are silenced during meiosis (Turner et al. 2005; Turner 2007; Cloutier and Turner 2010; Turner 2015). This suggests that the mode of MSCI varies across species and may depend on synapsed and unsynapsed chromosome dynamics during meiosis.

DC may coevolve with sex-biased gene expression of the X chromosome. Morabine grasshoppers show an excess of female-biased expression and a deficit of male-biased genes on the X and XL chromosomes. This feminization of the X (and/or XL) chromosome is most apparent in the gonads, while male-biased genes are enriched on autosomes and on XR-Y. Similar X chromosome feminization in flies (Sturgill et al. 2007; Vicoso and Bachtrog 2015) is linked to reduced testis expression, mediated by MSCI (Vibranovski et al. 2009; Mahadevaraju et al. 2021). The orthopteran X and/or XL chromosomes precociously condenses into a heterochromatic body (the so-called “sex chromatin”) very early during meiosis, specifically during the Leptotene stage (White 1973; Hewitt 1979; Castillo et al. 2010; Santos et al. 2024). This behavior of the hemizygous X and or XL chromosomes has long been interpreted as indirect evidence of MSCI (White 1978; Hewitt 1979). MSCI may further promote the demasculinization of the X and XL chromosomes and thus their feminization in morabine grasshoppers.

Our findings provide key insights into the evolution of DC, MSCI, and sex-biased gene expression in *V. viatica* grasshoppers. We demonstrate that DC operates differently in sex-specific reproductive tissues and in nonreproductive tissues: in non reproductive cells, X- and XL-linked genes are upregulated to balance expression between sexes, while in testes, MSCI presumably leads to transcriptional silencing of these chromosomes. This tissue-specific regulation likely reflects the distinct functional demands of each system, maintaining gene balance in soma while ensuring proper meiosis in germ cells. Additionally, we show that the X and XL chromosomes are enriched in female-biased genes, while the neo-XR-Y, including the SLR and PAR, retains ancestral autosomal gene expression. We anticipate that the progression of recombination suppression between XR and neo-Y, followed by the gradual degradation of the neo-Y, will follow a trajectory similar to that of the ancestral X chromosome in *Drosophila* and mammals. The observed demasculinization and feminization of the X and XL likely coevolves with MSCI, which suppresses testis-specific gene expression, a pattern seen across many groups of insects. The lack of MSCI on the XR-Y chromosome further supports the role of unsynapsed chromatin silencing in regulating X and XL hemizygous genes. Our findings highlight the complex interplay between MSCI, canonical DC, and sex-biased gene evolution and underscore the importance of future single-cell expression studies to clarify the extent of X and XL inactivation during meiosis.

## Materials and Methods

### Reference Genome Assemblies

To analyze sex-biased gene expression, we used the recently generated chromosome-level assemblies of the P24X0 and P24XY races as references for mapping RNA-seq data. The assemblies, generated from a previous study PRJNA945230 (Jayaprasad et al. 2024), are publicly available through NCBI under accession numbers JARYHR01 for P24X0 and JARYHQ01 for P24XY. These assemblies provide high-quality references that include complete sequences of both sex chromosomes and autosomes, facilitating accurate gene expression analysis in both races. The GFF annotation files used for gene expression analysis were also obtained from a previous study (Jayaprasad et al. 2024).

### RNA-Seq Data

RNA-seq reads were obtained from publicly available datasets on NCBI, accessible under the BioProject PRJNA668746 (Palacios-Gimenez et al. 2020). The RNA-seq data consisted of paired-end Illumina reads (2 × 150 bp) generated from male and female individuals of both P24X0 and P24XY races, covering multiple biological replicates for every tissue type (Table S1).

### RNA-Seq Read Processing and Mapping

Raw RNA-seq reads from all samples were quality-checked using FastQC (version 0.12.1) (Andrews 2010) and trimmed using TrimGalore (Version 0.6.10) (https://github.com/FelixKrueger/TrimGalore.git) with default settings to remove low-quality bases and adapter sequences. The trimmed reads were then mapped to their respective genome assemblies (P24X0 or P24XY) using STAR (version 2.7.2b) (Dobin et al. 2013) with paired-end alignment settings. The RNA-seq mapping statistics for P24X0 and P24XY samples are summarized in the Supplementary material (Table S1).

### Gene Expression Quantification

Aligned reads were processed using featureCounts from the Subread package v2.0.0 (Liao et al. 2019) in paired-end mode to quantify gene-level read counts. We provided featureCounts with GFF annotation file of the annotated genes, and per-genome counts were obtained using reads and lengths of the corresponding genes. To explore similarities in gene expression patterns within races, we performed sample-level quality control (QC) using principal component analysis (PCA). For this, we first transformed normalized read counts using the regularized logarithm (rlog) function implemented in DESeq2 (Love et al. 2014) in R v4.2.2 (R Core Team 2022). We identified three sample outliers in each race and therefore removed these prior to the gene expression analysis (Fig. S1). We used a custom R script to calculate TPM (Transcripts Per Million) values, given a normalized gene expression level. Only genes with TPM > 1 in each biological replicate were included in the analysis. The TPM values from replicates of each samples were averaged for the final plots of gene expression in DC analysis. We compared gene expression per chromosome type (i.e. autosomes, X chromosomes, XL and XR-Y arms including SLR and PAR), separately in adult gonads, heads and legs from male and female individuals.

We identified one-to-one orthologous genes across chromosome types in the races P24X0 and P24XY using bidirectional best BLASTp hits with an e-value threshold of < 1e-10. We then matched the one-to-one orthologous gene IDs with those in the gene expression matrices generated above to assess their expression profiles. For comparison, we retained only highly expressed genes (TPM > 1). To test for differences in gene expression, we applied the Kruskal–Wallis test for overall group differences, followed by pairwise Wilcoxon rank-sum tests with Benjamini–Hochberg correction for multiple comparisons using the ggstatsplot function (Patil 2021) in R.

### Differential Gene Expression Analysis

To identify sex-biased genes per chromosome type, differential expression analysis was performed using DESeq2 (Love et al. 2014) in R v4.2.2 (R Core Team 2022). Raw read counts served as input, and fold change (FC) values were calculated as the male-to-female expression ratio for each gene. All Wald test-based *P*-values were adjusted (*P*adj) for multiple testing. Genes were classified based on the following criteria:

*Male-biased genes*: Log2FC > 2 and *P*adj < 0.05.

*Female-biased genes*: Log2FC < -2 and *P*adj < 0.05.

*Unbiased genes*: Genes not meeting the above thresholds.

This pipeline allowed us to investigate patterns of sex-biased gene expression and to explore the evolution of dosage compensation mechanisms in *Vandiemenella* grasshoppers.

## Supplementary Material

evag026_Supplementary_Data

## Data Availability

The genome assemblies of P24 races are available under the BioProject PRJNA945230. The RNA-Seq reads are available under the BioProject PRJNA668746. The custom R scripts used for differential gene expression analyses and visualization are available at https://github.com/suvi93/VvRNA-analyses.
