# Supplementary material for: Evolutionary Dynamics of Dosage Compensation and Sex-biased Gene Expression in Morabine Grasshopper Vandiemenella viatica
Source: Genome Biol Evol. 2026 Feb 4;18(2):evag026. doi: 10.1093/gbe/evag026 (PMC12930189; doi:10.1093/gbe/evag026)
Supplement: evag026_Supplementary_Data [file evag026_supplementary_data.zip › Supplementary material.docx]

## Supplementary material

**Figure legends**

**Figure S1.** PCA-based quality control of P24X0 and P24XY RNA-seq samples, shown in the 2D space of the first two principal components. Asterisks (*) denote removed sample outliers in each plot.

**Tables**

**Table S1.** Summary of RNA-seq mapping statistics for P24X0 and P24XY samples.
