# Supplementary figures and images for: Evolutionary Dynamics of Dosage Compensation and Sex-biased Gene Expression in Morabine Grasshopper Vandiemenella viatica
Source: Genome Biol Evol. 2026 Feb 4;18(2):evag026. doi: 10.1093/gbe/evag026 (PMC12930189; doi:10.1093/gbe/evag026)

**(a) P24X0 – All Samples**

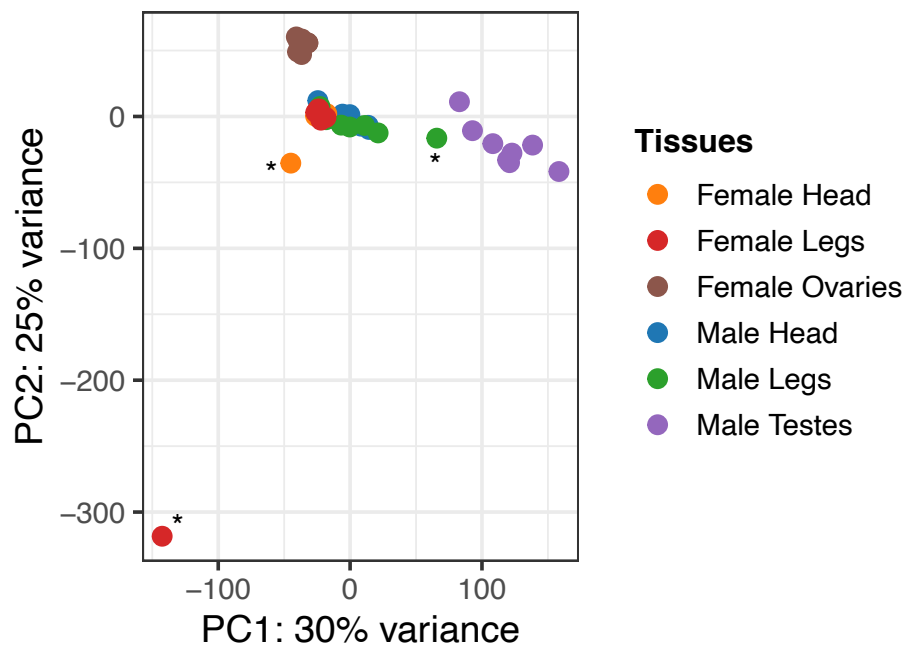

**(b) P24X0 – Outliers Removed**

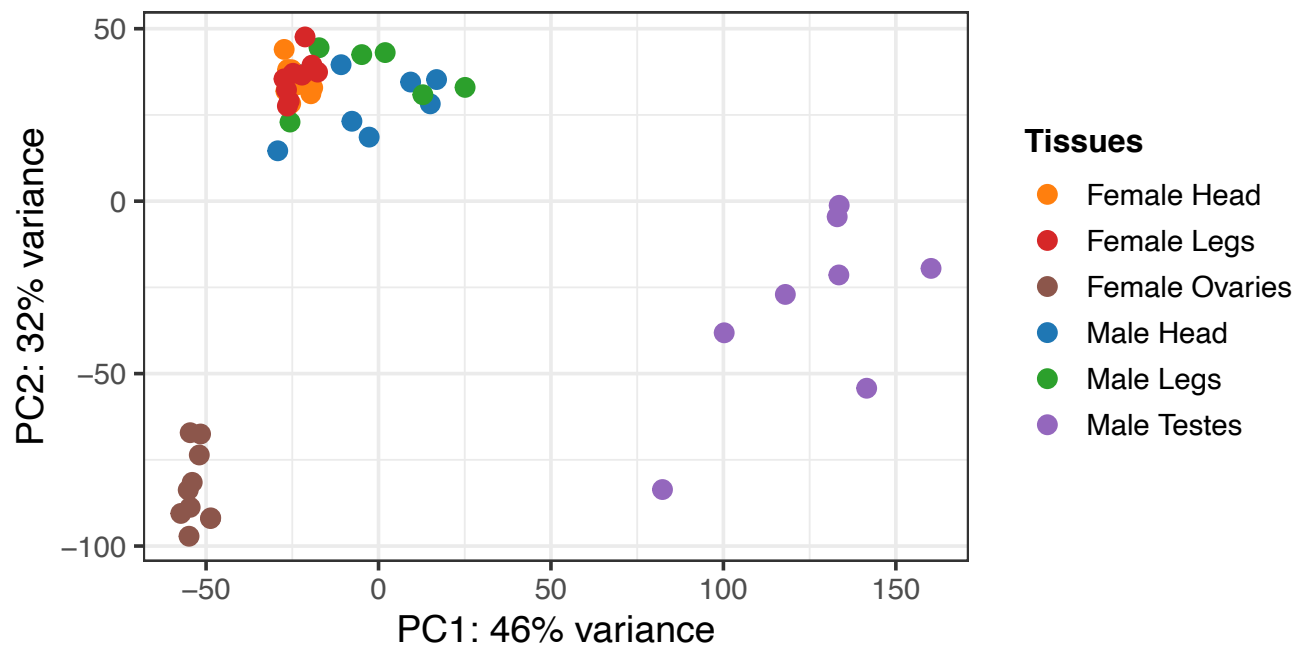

**(c) P24XY – All Samples**

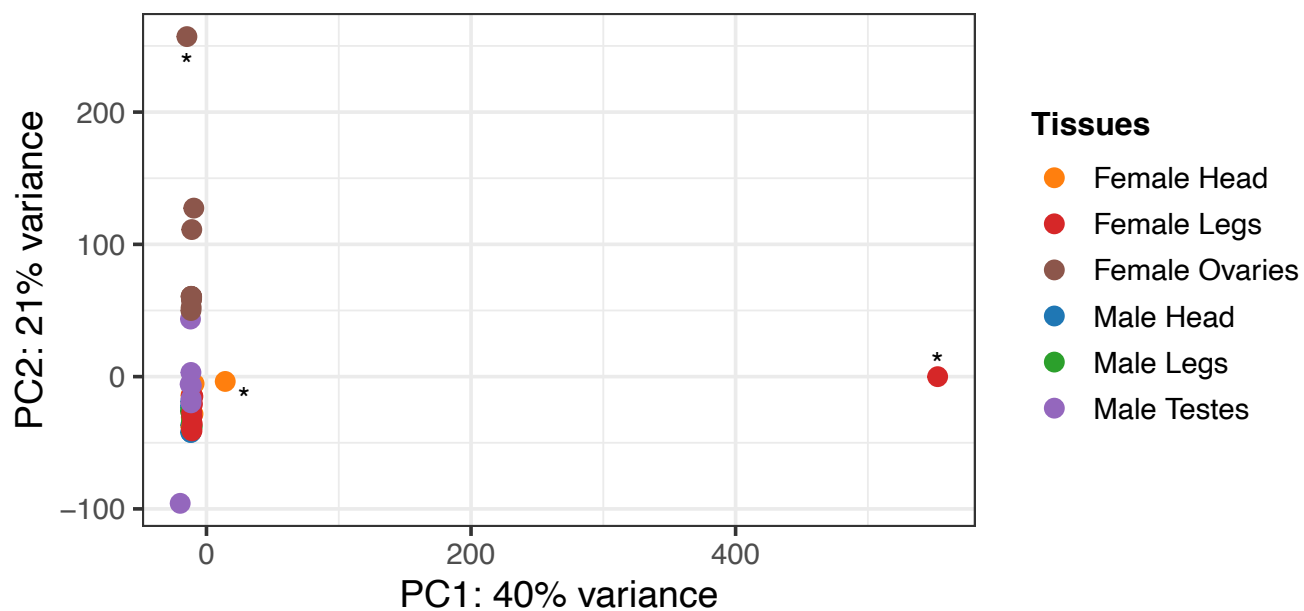

**(d) P24XY – Outliers Removed**

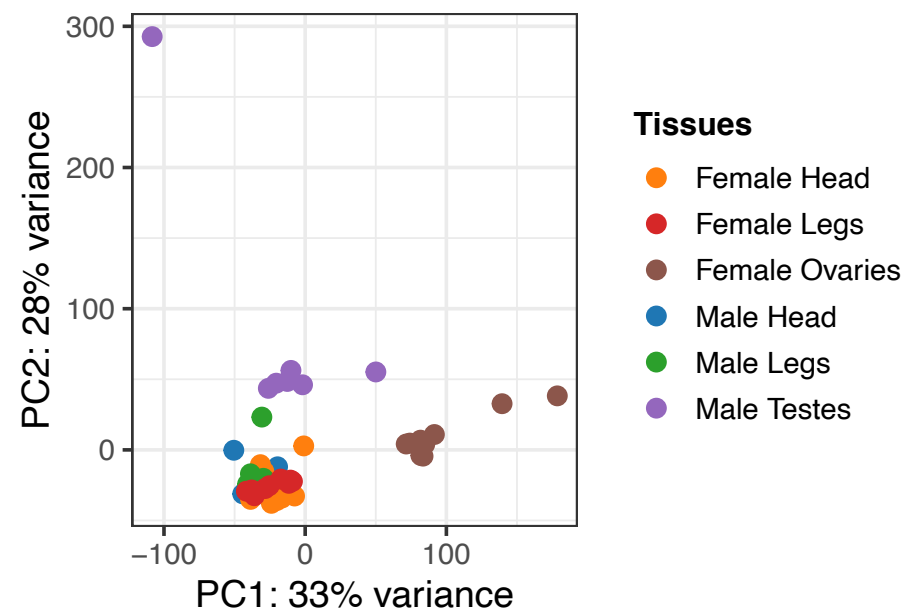

Supplement: evag026_Supplementary_Data [file evag026_supplementary_data.zip › Figure_SM1.pdf]
